# Increased CD9 expression predicts favorable prognosis in human cancers: a systematic review and meta-analysis

**DOI:** 10.1186/s12935-021-02152-y

**Published:** 2021-09-07

**Authors:** Hyun Min Koh, Bo Gun Jang, Dong Hui Lee, Chang Lim Hyun

**Affiliations:** 1 Department of Pathology, Gyeongsang National University Changwon Hospital, Changwon, Republic of Korea; 2 Department of Pathology, Jeju National University School of Medicine, 15 Aran 13-gil, Jeju, 63241 Republic of Korea; 3 Department of Pathology, Jeju National University Hospital, Jeju, Republic of Korea

**Keywords:** Cancer, CD9, Meta-analysis, Prognosis, Survival

## Abstract

**Background:**

CD9 is implicated in cancer progression and metastasis by its role in suppressing cancer cell proliferation and survival. However, the prognostic and clinicopathological significance of CD9 expression is controversial. Therefore, the current meta-analysis was conducted to determine the prognostic and clinicopathological significance of CD9 expression in cancer patients.

**Methods:**

Eligible studies were selected through database search of PubMed, Embase and Cochrane library up to April 5 2020. The necessary data were extracted from the included studies. Pooled hazard ratio (HR) and odds ratio (OR) with 95% confidence interval (CI) were calculated to evaluate the prognostic and clinicopathological significance of CD9 expression in cancer patients.

**Results:**

A total of 17 studies consisting of 3456 cancer patients were included in this meta-analysis. An increased CD9 expression was significantly associated with a more favorable overall survival (OS) (HR 0.47, 95% CI 0.31–0.73, *p* = 0.001) and disease-free survival (DFS) (HR 0.48, 95% CI 0.30–0.79, *p* = 0.003). In subgroup analysis of cancer type, an increased CD9 expression was associated with increased OS in breast cancer and digestive system cancer, and with increased DFS in head and neck cancer and leukemia/lymphoma. Additionally, an increased CD9 expression significantly correlated with lower overall stage (OR 0.45, 95% CI 0.29–0.72, *p* = 0.001).

**Conclusion:**

An increased CD9 expression was associated with favorable survival in cancer patients suggesting that CD9 expression could be a valuable survival factor in cancer patients.

**Supplementary Information:**

The online version contains supplementary material available at 10.1186/s12935-021-02152-y.

## Background

Cancer is a crucial public health problem worldwide [1]. The incidence of cancer is rising worldwide due to an increase in the elderly population and the increasing prevalence of cancer risk factors such as obesity, smoking, and changing reproductive patterns [2]. Despite remarkable progress in cancer treatment, many cancer patients die from cancer recurrence and progression. Therefore, studies have recently been conducted on a variety of biomarkers that can measure the prognosis of cancer patients and predict treatment effects, and it is imperative to identify novel markers that predict the progression and prognosis of cancer as well as the treatment outcome.

There are at least 33 human tetraspanin proteins that have important functions in the cancer, immune system and cellular signaling [3]. Tetraspanins are membrane proteins with four transmembrane domains, such as CD9, CD37, CD53, CD81, CD82, and CD152 [3, 4]. CD9 is a member of the tetraspanin family and is widely expressed on the surface of various cells, including cancer cells as well as normal epithelial, endothelial, and hematopoietic cells [3]. CD9 is involved in biological and pathological processes, interacting with a variety of cell-surface molecules, including integrins, growth factor receptor, transmembrane proteins, and signaling molecules [5, 6]. CD9 plays a role in cellular adhesion, motility, proliferation, survival, and fertilization and is reported as a key player in the development of cancer [3]. CD9 has also been used in human cancer processes, such as metastasis or angiogenesis [7].

Experimental studies using cancer cells show that CD9 basically interrupts the progression and metastasis of cancer by suppression of cancer cell proliferation and survival [3]. Accumulating data have reported that CD9 was associated with a favorable prognosis in breast [8, 9], colorectal [10, 11], esophageal [12], gallbladder [13], pancreatic [14], head and neck [15, 16], and lung cancer [17], and urothelial carcinoma [18], mesothelioma [19], and hematologic malignancy [7, 20]. Nevertheless, some studies suggest contrary results [6, 21, 22]. Furthermore, published studies report small sample sizes and individual results, making it difficult to comprehensively evaluate the association between CD9 expression and prognosis in cancer patients. Therefore, the current meta-analysis was conducted to comprehensively elucidate the prognostic and clinicopathological significance of CD9 expression in cancer patients.

The following section will describe search strategy, selection criteria, data extraction, quality assessment of included studies, and statistical analysis. The results section will describe search results, main characteristics of included studies, the association between CD9 expression and survival, and the association between CD9 expression and clinicopathological characteristics. Additionally, the publication bias and sensitivity test will be described.

## Methods

### Search strategy

This study was conducted according to the preferred reporting items for systematic reviews and meta-analyses (PRISMA) statement [2]. Relevant studies, up to those from April 5, 2020, were identified by searching PubMed, Embase, and the Cochrane Library databases using the terms (CD9) and (cancer, tumor, carcinoma, neoplasm, or malignancy) and (prognostic, predict, prognosis, survival, or outcome). All significant publications in the references of the reviewed articles were manually searched to identify qualified articles.

### Inclusion and exclusion criteria

The included studies complied with the following criteria: (i) CD9 expression was detected in human cancer cells, (ii) the association between CD9 expression and clinical outcome was assessed, and (iii) the hazard ratio (HR) and 95% confidence interval (CI) for clinical outcomes were provided. The excluded articles were (i) duplicate studies; (ii) conference abstracts, case reports, reviews, letters, and non-English articles; and (iii) preclinical studies, such as laboratory or in vitro studies.

### Data extraction

The basic data extracted from each study included the first author, publication year, country, cancer type, sample size, patient’s sex, mean or median age, study period, follow-up period, clinical outcome, detection method and a cut-off value of CD9 expression, and survival analysis method. Overall survival (OS), disease-free survival (DFS), progression-free survival, and recurrence-free survival were regarded as endpoints [2]. HRs with CIs that were directly provided were used to estimate the association between CD9 expression and prognosis in cancer patients. The data were individually extracted by two authors, and discordances were resolved by reaching a consensus.

### Quality assessment

The quality of each study was evaluated using the Newcastle–Ottawa Scale (NOS). The NOS consists of three categories: Selection, Comparability, and Outcome. The criteria of NOS were as follows: Selection (i) representativeness of the exposed cohort, (ii) selection of the non-exposed cohort, (iii) ascertainment of exposure, (iv) demonstration that outcome of interest was not present at start of study; Comparability (i) comparability of cohorts on the basis of the design or analysis; and Outcome (i) assessment of outcome, (ii) was follow-up long enough for outcomes to occur, (iii) adequacy of follow up of cohorts [23]. The NOS scores ranged from 0 to 9. Studies with a score of 6 or more were regarded as good quality studies. Two authors independently evaluated the quality of the included studies.

### Statistical analyses

StataSE12 (Stata, College Station, TX, USA) was used for quantitative assessment. The pooled HR with 95% CI was calculated to determine the association of CD9 expression and prognosis, and the pooled odds ratio (OR) with 95% CI was assessed for investigating the association of CD9 expression and clinicopathological characteristics in cancer patients. I^2^ statistics were used to evaluate heterogeneity among the included studies. The random-effects model was applied when *p*–value was < 0.05 or the I^2^ value was > 50%, otherwise the fixed-effects model was applied. Subgroup analysis and meta-regression were performed to reveal the cause of heterogeneity. Funnel plots and Egger tests were also conducted to check for publication bias. Sensitivity analysis was performed to confirm consistency of the pooled results. A p-value < 0.05 was considered statistically significant.

## Results

### Search results

**Fig. 1:**
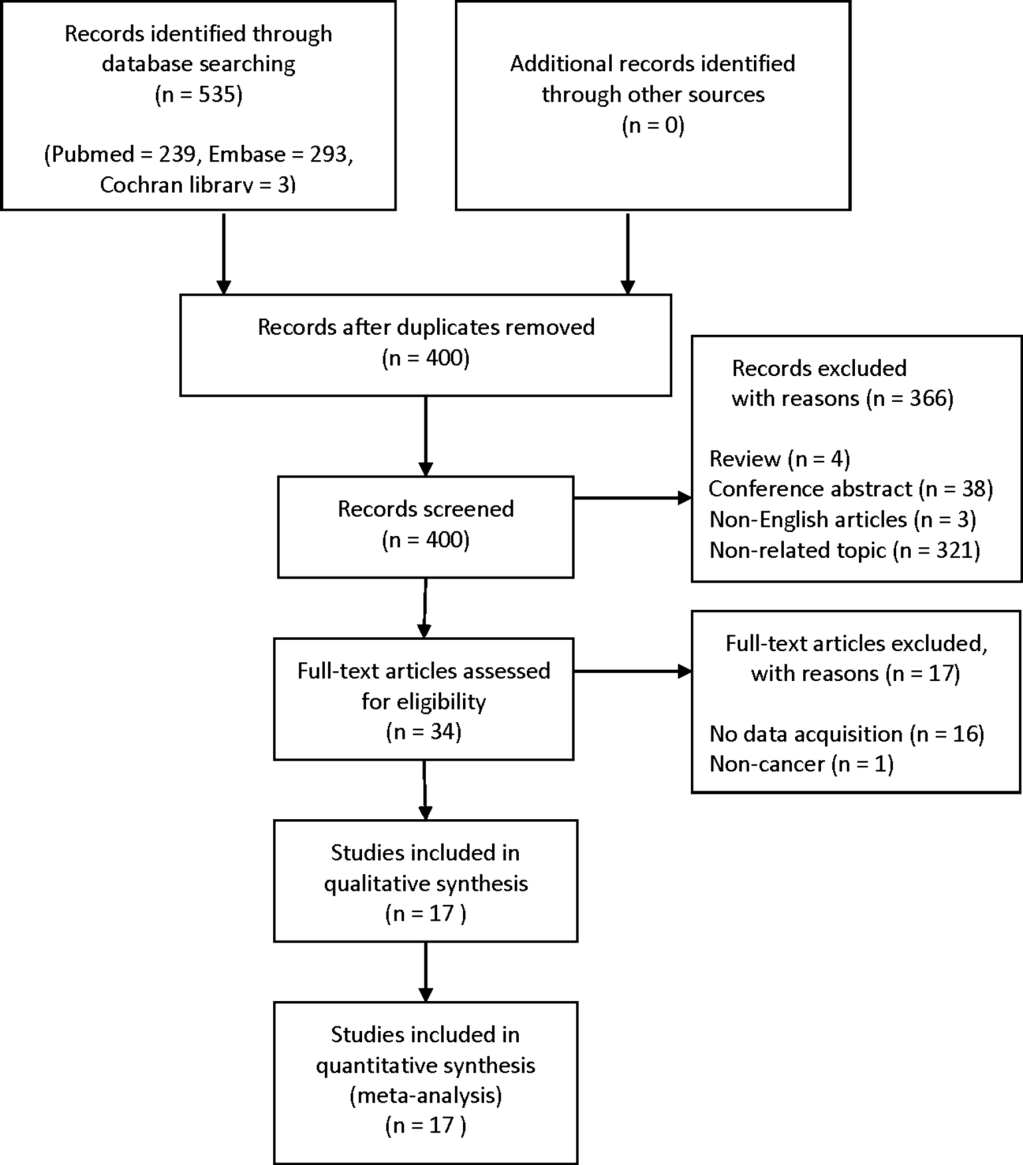
Flow diagram of the study selection process

As shown Fig. 1, 535 studies were searched from PubMed, Embase, and Cochrane library using the search strategy described above. Among them, 135 studies were excluded due to duplicates. Next 366 studies were excluded because they were review articles, conference abstracts, non-English articles, and non-related topics. Subsequently, the remaining 34 studies were assessed to exclude articles that did not provide relevant data, and finally 17 studies were included in this meta-analysis.

### Main characteristics of the included studies

The main characteristics of the studies included are shown in Table 1. The total number of studies was 17 consisting 3456 cancer patients with 34 being minimum sample size, and 1349 being the largest. The studies were published from 1995 to 2019. Seven studies were conducted in Japan, and three each in South Korea and China. Two studies were in Switzerland, and one each were in France and Austria. The types of cancer assessed in this meta-analysis were breast cancer (n = 4), colorectal cancer (n = 2), esophageal cancer (n = 1), gallbladder cancer (n = 1), pancreatic cancer (n = 1), head and neck cancer (n = 2), lung cancer (n = 1), urothelial carcinoma (n = 1), leukemia (n = 2), lymphoma (n = 1), and malignant mesothelioma (n = 1). As for detection method of CD9 expression, 12 used immunohistochemistry (IHC) tests, 3 used reverse transcription-polymerase chain reaction (RT-PCR), and 2 used flow cytometry to assess CD9 expression in the cancer cells. The included studies had NOS scores ranging from 6 to 8, indicating high quality.

**Table 1:** Main characteristics of the included studies in this meta-analysis

Study	Country	Cancer type	Sample size	Sex of patients (male /female)	Mean or median age (years)	Study period	Mean or median follow-up (months)	Clinical outcome	CD9 detection	Cut-off value of CD9 expression	Survival analysis	NOS
Baek et al. [6]	South Korea	Invasive lobular carcinoma	113	NA	48 (27–88)	2001–2013	62 (7–187)	DFS	IHC	Staining scores with intensity and proportion (≥ 4)	MVA	8
Touzet et al. [7]	France	Acute myeloid leukemia	112	61/51	51.9 (20.9–77.6)	2009–2016	NA	OS, RFS	Flow cytometry	20% of all the blasts	UVA(OS) MVA(RFS)	7
Liang et al. [22]	China	Acute lymphoblastic leukemia	112	48/64	39.46	2013–2016	NA	OS	Flow cytometry	20% of all the blasts	MVA	7
Kwon et al. [21]	South Korea	Invasive breast cancer	1349	NA	NA	1995–2007	115 (1–233)	DFS	IHC	Staining scores with intensity and proportion (4)	MVA	8
Kim et al. [10]	South Korea	Colorectal carcinoma	304	163/141	NA	1996–2000	4–243	DFS	IHC	Staining scores with intensity and proportion (3)	MVA	8
Dong et al. [20]	China	Follicular lymphoma	76	32/44	50.90 (25–76)	2006–2013	NA	PFS	IHC	Staining scores with intensity and proportion (3)	UVA	6
Amatya et al. [19]	Japan	Malignant mesothelioma	112	103/9	65.8 (42–88)	NA	16.5 (0–79)	OS	IHC	Negative: no staining	MVA	8
Zou et al. [13]	China	Gallbladder adenocarcinoma	108	24/84	52.6 (35–70)	1996–2006	NA	OS	IHC	25%	MVA	7
Mhawech et al. [15]	Switzerland	Head and neck squamous cell carcinoma	153	122/31	59 (35–90)	1992–1999	65 (14–123)	DFS	IHC	50%	MVA	8
Hashida et al. [11]	Japan	Colon cancer	146	84/62	62.8 (35–80)	1994–2001	44.3 (6.3–85.9)	OS, DFS	IHC	Staining scores with intensity and proportion (120)	MVA	8
Erovic et al. [16]	Austria	Head and neck squamous cell carcinoma	34	27/7	56 (42–77)	1985–2001	0–92	OS, DFS	IHC	50%	UVA(OS) MVA(DFS)	8
Mhawech et al. [18]	Switzerland	Urothelial bladder carcinoma	320	257/63	70.4 (27–98)	1988–1999	43.6 (112–240)	RFS	IHC	5%	MVA	8
Shimada et al. [12]	Japan	Eaophageal squamous cell carcinoma	116	98/18	63.9	1987–1995	5.69	OS	IHC	50%	MVA	8
Huang et al. [9]	Japan	Breast cancer	109	NA	NA	1987–1995	48.5	OS, DFS	RT-PCR	Positive/Negative	MVA	8
Sho et al. [14]	Japan	Pancreatic cancer	40	31/9	66 (47–80)	1992–1997	22.9 (5–62)	OS	RT-PCR	Positive/Decreased	MVA	8
Miyake et al. [8]	Japan	Breast cancer	143	NA	NA	1990–1992	45.7 (30–61)	OS, DFS	IHC	50%	MVA	8
Higashiyama et al. [17]	Japan	Non-small cell lung cancer	109	80/29	NA	1991–1992	43.7 (31–55)	OS	RT-PCR	Positive/Negative	MVA	8

*DFS* disease-free survival, *IHC* immunohistochemistry, *MVA* multivariate analysis, *NA* not available, *NOS* Newcastle–Ottawa Scale, *OS* overall survival, *PFS* progression-free survival, *RFS* recurrence-free survival, *RT-PCR* reverse transcription-polymerase chain reaction, *UVA* univariate analysis

### Association between CD9 expression and OS

**Fig. 2:**
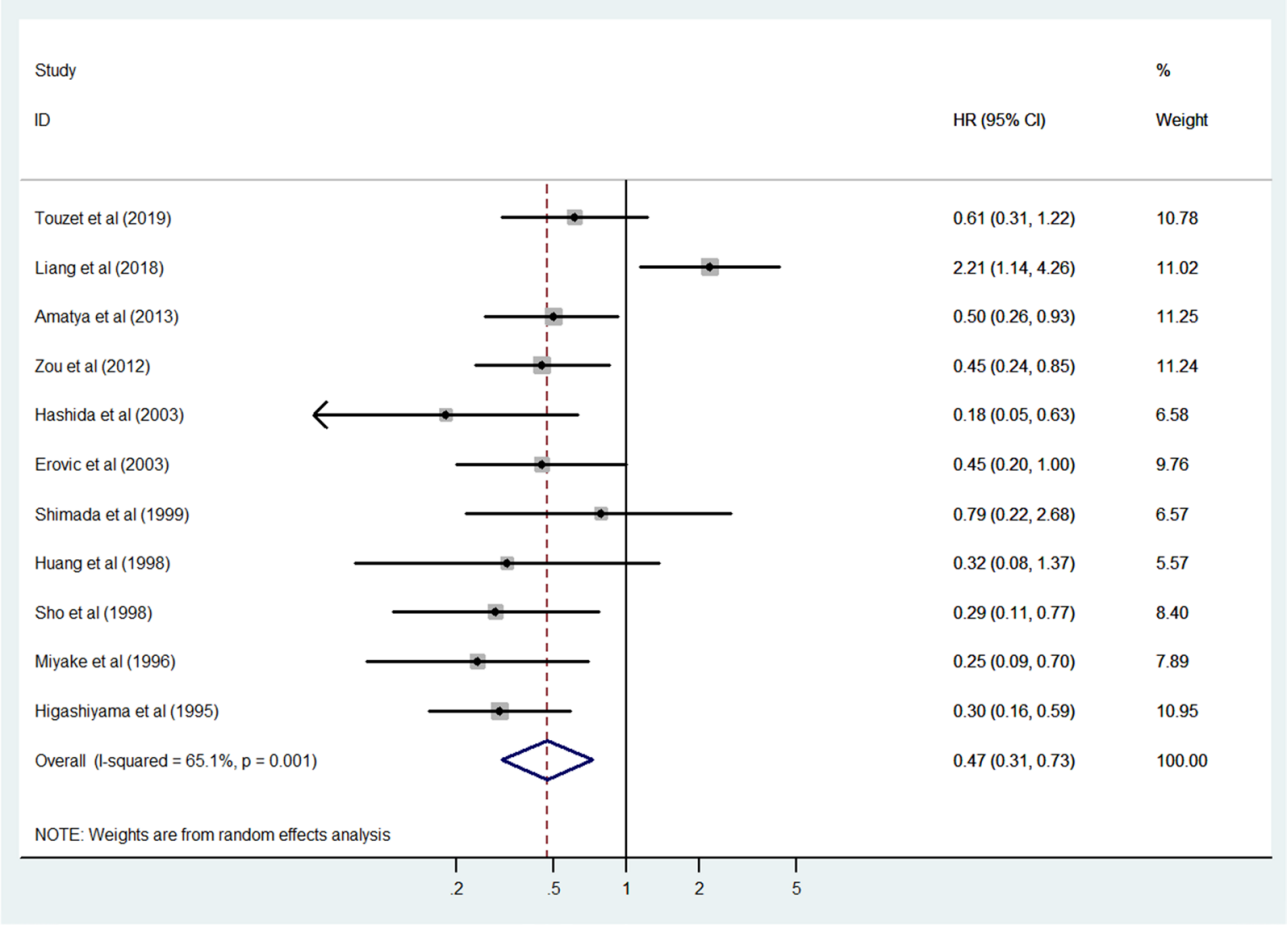
Forest plot of the association between CD9 expression and overall survival in human cancers

**Fig. 3:**
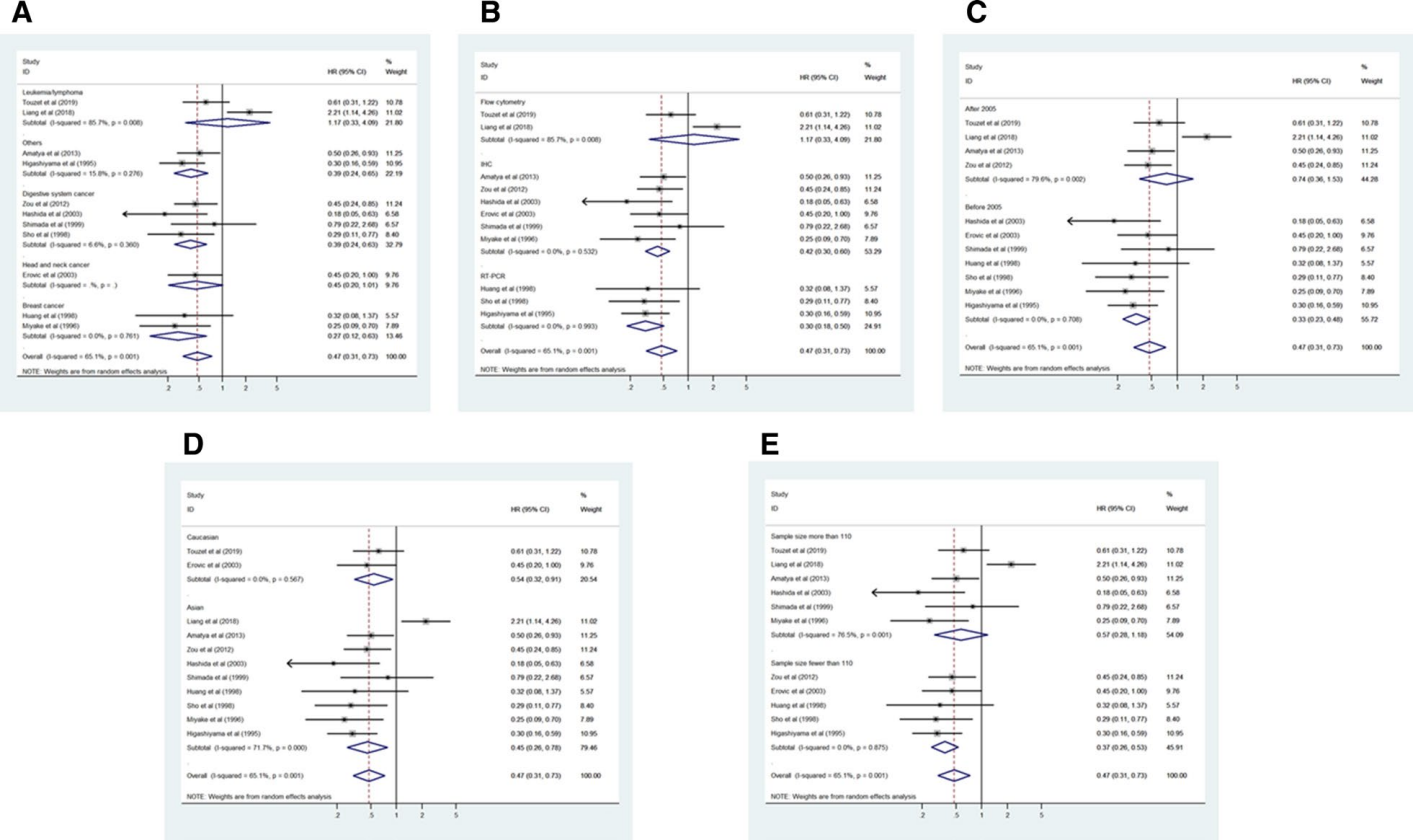
Forest plot of subgroup analysis of the association between CD9 expression and overall survival in human cancers. **A** Subgroup analysis stratified by cancer type, **B** subgroup analysis stratified by CD9 detection method, **C** subgroup analysis stratified by publication year, **D** subgroup analysis stratified by race, and **E** subgroup analysis stratified by sample size

Eleven studies consisting of 1141 cancer patients reported an association between CD9 expression and OS. The pooled HR was calculated using a random-effects model because of the significant heterogeneity between included studies (I^2^ = 65.1%, *p* = 0.001). The pooled HR was 0.47 (95% CI 0.31–0.73, *p* = 0.001), indicating an association between increased CD9 expression and favorable OS in cancer patients (Fig. 2). To investigate the source of significant heterogeneity among these studies, subgroup analysis was performed based on the cancer type, CD9 detection method, publication year, race, and sample size (Fig. 3A–E). The results of the subgroup analysis revealed that the association between increased CD9 expression and favorable OS was still significant in all of the above factors (breast cancer, HR 0.27, 95% CI 0.12–0.63, *p* = 0.002; digestive system cancer, HR 0.39, 95% CI 0.24–0.63, *p* < 0.001; other cancers, HR 0.39, 95% CI 0.24–0.65, *p* < 0.001; IHC, HR 0.42, 95% CI 0.30–0.60, *p* < 0.001; RT-PCR, HR 0.30, 95% CI 0.18–0.50, *p* < 0.001; published studies before 2005, HR 0.33, 95% CI 0.23–0.48, *p* < 0.001; Asian, HR 0.45, 95% CI 0.26–0.78, *p* = 0.004; Caucasian, HR 0.54, 95% CI 0.32–0.91, *p* = 0.020; sample size < 110, HR 0.37, 95% CI 0.26–0.53, *p* < 0.001) except in the subgroup with head and neck cancer (HR 0.45, 95% CI 0.20–1.01, *p* = 0.052) and leukemia/lymphoma (HR 1.17, 95% CI 0.33–4.09, *p* = 0.809), the subgroup where CD9 expression was detected using flow cytometry (HR 1.17, 95% CI 0.33–4.09, *p* = 0.809), the subgroup with publications after 2005 (HR 0.74, 95% CI 0.36–1.53, *p* = 0.417), and the subgroup where the sample size was > 110 (HR 0.57, 95% CI 0.28–1.18, *p* = 0.131) (Table 2). To further determine the cause of heterogeneity, meta-regression was conducted with covariates that also consisted of the above factors including cancer type, CD9 detection method, publication year, race, and sample size. Meta-regression analysis suggested that the CD9 detection method (*p* = 0.015) was likely to be the cause of heterogeneity (Table 2).

**Table 2:** Subgroup analysis and meta-regression of the association between CD9 expression and overall survival in cancer patients

Subgroup	Number of studies	Number of patients	Pooled HR (95% CI)	p value	Heterogeneity		Meta-regression
					I^2^ (%)	p value	p value
Cancer type							0.298
Breast cancer	2	252	0.27 (0.12–0.63)	0.002	0.0	0.761	
Digestive system cancer	4	410	0.39 (0.24–0.63)	< 0.001	6.6	0.360	
Head and neck cancer	1	34	0.45 (0.20–1.01)	0.052	-	-	
Leukemia/lymphoma	2	224	1.17 (0.33–4.09)	0.809	85.7	0.008	
Others	2	221	0.39 (0.24–0.65)	< 0.001	15.8	0.276	
CD9 detection method							0.015
Flow cytometry	2	224	1.17 (0.33–4.09)	0.809	85.7	0.008	
IHC	6	659	0.42 (0.30–0.60)	< 0.001	0.0	0.532	
RT-PCR	3	258	0.30 (0.18–0.50)	< 0.001	0.0	0.993	
Publication year							0.060
After 2005	4	444	0.74 (0.36–1.53)	0.417	79.6	0.002	
Before 2005	7	697	0.33 (0.23–0.48)	< 0.001	0.0	0.708	
Race							0.804
Asian	9	995	0.45 (0.26–0.78)	0.004	71.7	< 0.001	
Caucasian	2	146	0.54 (0.32–0.91)	0.020	0.0	0.567	
Sample size							0.257
Fewer than 110	5	400	0.37 (0.26–0.53)	< 0.001	0.0	0.875	
More than 110	6	741	0.57 (0.28–1.18)	0.131	76.5	0.001	

*CI* confidence interval, *HR* hazard ratio, *IHC* immunohistochemistry, *RT-PCR* reverse transcription-polymerase chain reaction

### Association between CD9 expression and DFS

**Fig. 4:**
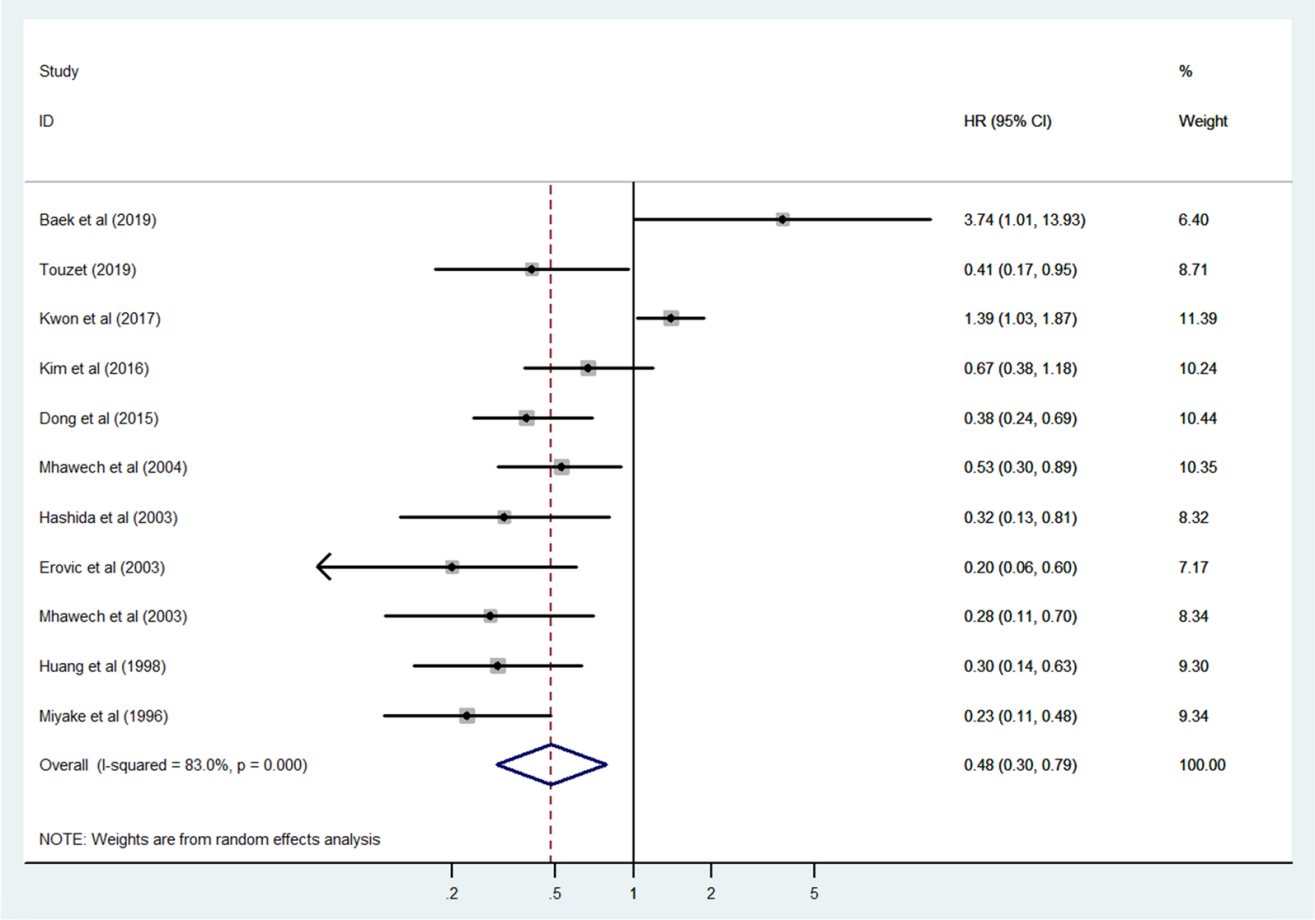
Forest plot of the association between CD9 expression and disease-free survival in human cancers

**Fig. 5:**
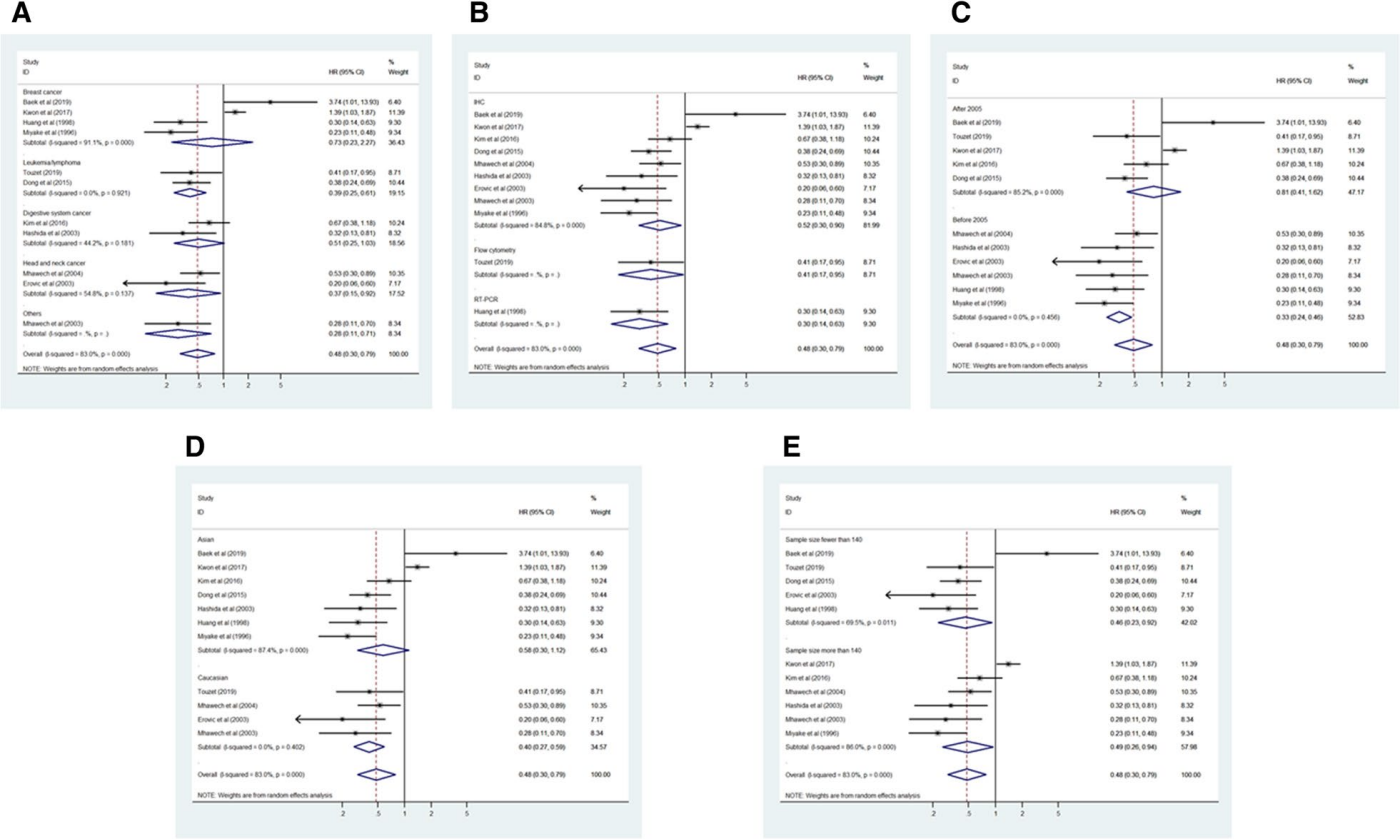
Forest plot of subgroup analysis of the association between CD9 expression and disease-free survival in human cancers. **A** Subgroup analysis stratified by cancer type, **B** subgroup analysis stratified by CD9 detection method, **C** subgroup analysis stratified by publication year, **D** subgroup analysis stratified by race, and **E** subgroup analysis stratified by sample size

Eleven studies consisting of 2859 cancer patients reported an association between CD9 expression and DFS, progression-free survival, and recurrence-free survival. In this meta-analysis, progression-free survival and recurrence-free survival were regarded as DFS. The pooled HR was assessed using a random-effects model due to significant heterogeneity (I^2^ = 83.0%, *p* = 0.003). The pooled HR was 0.48 (95% CI 0.30–0.79, *p* = 0.003), indicating a significant association between increased CD9 expression and favorable DFS in cancer patients (Fig. 4). Subgroup analysis was performed to explore the cause of heterogeneity according to the cancer type, CD9 detection method, publication year, race, and sample size (Fig. 5A–E). The results of subgroup analysis demonstrated that the association between an increased CD9 expression and favorable DFS remained statistically significant in all factors (head and neck cancer, HR 0.37, 95% CI 0.15–0.92, *p* = 0.033; leukemia/lymphoma, HR 0.39, 95% CI 0.25–0.61, *p* < 0.001; other cancers, HR 0.28, 95% CI 0.11–0.71, *p* = 0.007; flow cytometry, HR 0.41, 95% CI 0.17–0.95, *p* = 0.039; IHC, HR 0.52, 95% CI 0.30–0.90, *p* = 0.020; RT-PCR, HR 0.30, 95% CI 0.14–0.63, *p* = 0.002; published studies before 2005, HR 0.33, 95% CI 0.24–0.46, *p* < 0.001; Caucasian, HR 0.40, 95% CI 0.27–0.59, *p* < 0.001; sample size < 140, HR 0.46, 95% CI 0.23–0.92, *p* = 0.028; sample size > 140, HR 0.49, 95% CI 0.26–0.94, *p* = 0.032) apart from the subgroup with breast cancer (HR 0.73, 95% CI 0.23–2.27, *p* = 0.583) and digestive system cancer (HR 0.51, 95% CI 0.25–1.03, *p* = 0.059), the subgroup with publications after 2005 (HR 0.81, 95% CI 0.41–1.62, *p* = 0.552) and the Asian race subgroup (HR 0.58, 95% CI 0.30–1.12, *p* = 0.102) (Table 3). To further evaluate the source of heterogeneity, meta-regression was performed through covariates using the above factors including cancer type, CD9 detection method, publication year, race, and sample size. The result of meta-regression suggested that publication year (*p* = 0.033) was likely to be the source of heterogeneity (Table 3).

**Table 3:** Subgroup analysis and meta-regression of the association between CD9 expression and disease-free survival in cancer patients

Subgroup	Number of studies	Number of patients	Pooled HR (95% CI)	p value	Heterogeneity		Meta-regression
					I^2^ (%)	p value	p value
Cancer type							0.240
Breast cancer	4	1714	0.73 (0.23–2.27)	0.583	91.1	< 0.001	
Digestive system cancer	2	450	0.51 (0.25–1.03)	0.059	44.2	0.181	
Head and neck cancer	2	187	0.37 (0.15–0.92)	0.033	54.8	0.137	
Leukemia/lymphoma	2	188	0.39 (0.25–0.61)	< 0.001	0.0	0.921	
Others	1	320	0.28 (0.11–0.71)	0.007	–	–	
CD9 detection method							0.792
Flow cytometry	1	112	0.41 (0.17–0.95)	0.039	–	–	
IHC	9	2638	0.52 (0.30–0.90)	0.020	84.8	< 0.001	
RT-PCR	1	109	0.30 (0.14–0.63)	0.002	–	–	
Publication year							0.033
After 2005	5	1954	0.81 (0.41–1.62)	0.552	85.2	< 0.001	
Before 2005	6	905	0.33 (0.24–0.46)	< 0.001	0.0	0.456	
Race							0.354
Asian	7	2240	0.58 (0.30–1.12)	0.102	87.4	< 0.001	
Caucasian	4	619	0.40 (0.27–0.59)	< 0.001	0.0	0.402	
Sample size							0.896
Fewer than 140	5	444	0.46 (0.23–0.92)	0.028	69.5	0.011	
More than 140	6	2415	0.49 (0.26–0.94)	0.032	86.0	< 0.001	

CI, confidence interval; HR, hazard ratio; IHC, immunohistochemistry; RT-PCR, reverse transcription-polymerase chain reaction

### Association between CD9 expression and clinicopathological characteristics

An increased CD9 expression was significantly associated with patients’ sex (OR 0.76, 95% CI 0.59–0.99, *p* = 0.044) and lower overall stage (OR 0.45, 95% CI 0.29–0.72, *p* = 0.001). CD9 expression tended to increase in patients with smaller tumor size (OR 0.83, 95% CI 0.44–1.55, *p* = 0.554), lower tumor grade and stage (OR 0.50, 95% CI 0.16–1.60, *p* = 0.244; OR 0.56, 95% CI 0.27–1.16, *p* = 0.120), and absent lymph node metastasis (OR 0.68, 95% CI 0.41–1.13, *p* = 0.137); however, this increase was not statistically significant (Table 4, Additional file 1: Figures S1–S7).

**Table 4:** Association between CD9 expression and clinicopathological characteristics in cancer patients

Characteristic	Number of studies	Number of patients	Pooled OR (95% CI)	p value	Heterogeneity		
					I^2^ (%)	p value	Model
Age (old vs young)	10	2500	1.04 (0.88–1.22)	0.670	23.0	0.232	Fixed
Sex (male vs female)	9	1119	0.76 (0.59–0.99)	0.044	0.0	0.815	Fixed
Tumor size (large vs small)	3	1570	0.83 (0.44–1.55)	0.554	68.5	0.042	Random
Tumor grade (high vs low)	6	2340	0.50 (0.16–1.60)	0.244	94.9	< 0.001	Random
Tumor stage (high vs low)	8	2524	0.56 (0.27–1.16)	0.120	89.5	< 0.001	Random
Lymph node metastasis (present vs absent)	8	2312	0.68 (0.41–1.13)	0.137	83.0	< 0.001	Random
Overall stage (high vs low)	7	930	0.45 (0.29–0.72)	0.001	57.6	0.028	Random

*CI* confidence interval, *OR* odds ratio

### Publication bias

**Fig. 6:**
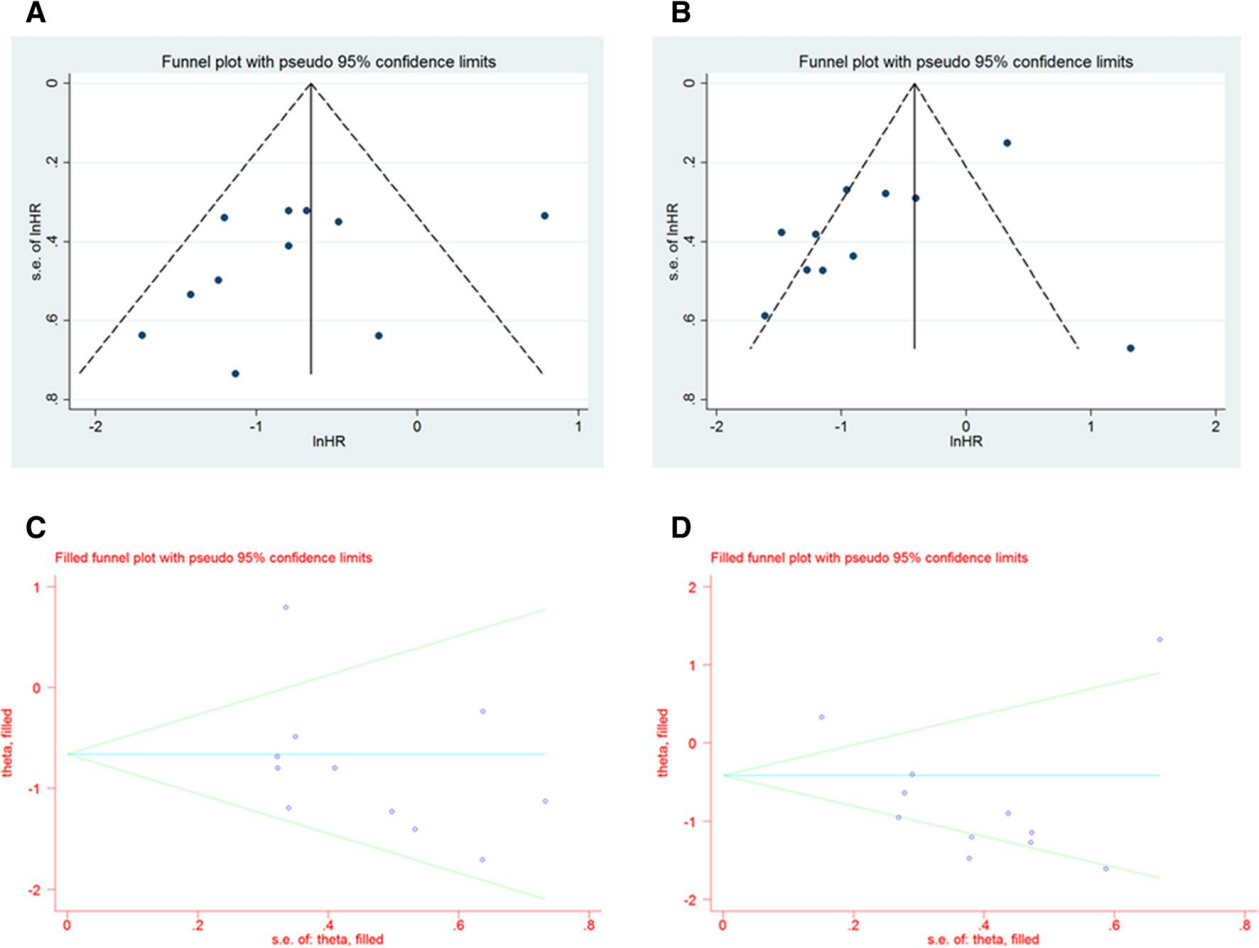
Funnel plot and trim and fill method for publication bias. **A** Funnel plot for overall survival, **B** funnel plot for disease-free survival, **C** trim and filled method for overall survival, **D** trim and filled method for disease-free survival

Visual inspection of the funnel plot showed asymmetry (Fig. 6A–B), which suggests the possibility of publication bias. Next, the Egger’s regression test was conducted. The result of Egger’s test was not statistically significant (*p* = 0.267) for OS but was significant for DFS (*p* = 0.039). The trim and fill method was also applied. The pooled results remained unchanged, indicating a significant association between CD9 expression, OS, and DFS in cancer patients (for OS, HR 0.47, 95% CI 0.31–0.73, *p* = 0.001; for DFS, HR 0.48, 95% CI 0.30–0.79, *p* = 0.003) (Fig. 6C–D).

### Sensitivity analysis

**Fig. 7:**
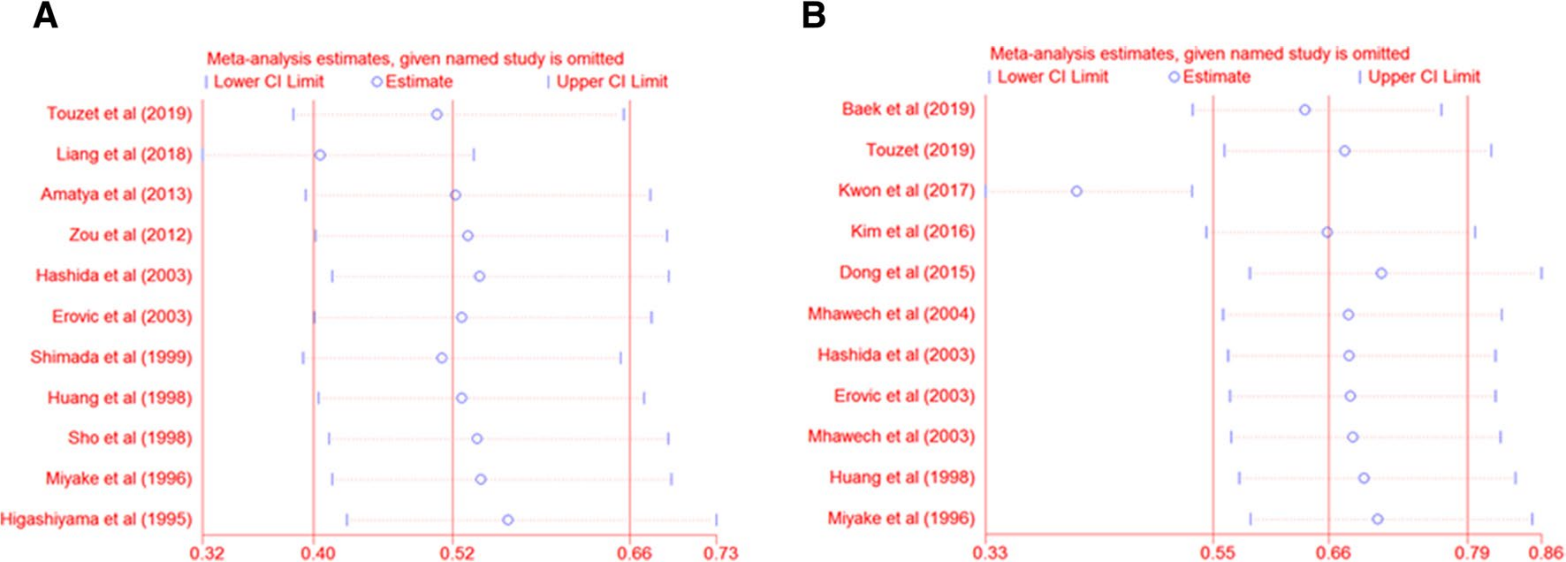
Sensitivity analysis of each included study. **A** Sensitivity analysis for overall survival, **B** sensitivity analysis for disease-free survival

Sensitivity analysis suggested that results from Liang et al. [22] and Kwon et al. [21] had significant effects on OS (HR 0.41, 95% CI 0.32–0.53) and DFS (HR 0.42, 95% CI 0.33–0.53), respectively. However, the pooled HR was not significantly changed after omitting individual articles. This indicated that our results were consistent and reliable (for OS, HR 0.52, 95% CI 0.40–0.66; for DFS, HR 0.66, 95% CI 0.55–0.79) (Fig. 7A–B).

## Discussion

As we know, this is the first systematic review and meta-analysis that demonstrated the prognostic significance of CD9 expression in human cancers. In the current study, 17 studies with a total of 3456 cancer patients were systematically analyzed. Our results indicated that increased CD9 expression was significantly associated with favorable OS and DFS in cancer patients. Additionally, subgroup analysis and meta-regression were conducted to explore the causes of heterogeneity. The outcomes of subgroup analysis revealed that the prognostic significance of CD9 expression changed with respect to the cancer type (head and neck cancer and leukemia/lymphoma), CD9 expression detection method (flow cytometry), publication year (after 2005), and sample size (> 110) for OS, as well as cancer type (breast cancer and digestive system cancer), publication year (after 2005), and race (Asian) for DFS. Furthermore, meta-regression analysis identified the detection method and publication year as the causes of the heterogeneity for OS and DFS, respectively. Regarding the clinicopathological characteristics, our analysis showed that an increased CD9 expression was significantly associated with the patient’s sex and lower overall stage. However, no significant association was shown between CD9 expression with tumor size, grade and stage, and lymph node metastasis. This allows us to infer that CD9 expression is more closely related to distant metastasis that to tumor stage or lymph node metastasis, which may affect prognosis in cancer patients. Indeed, previous studies have reported that decreased CD9 expression is generally related to more venous invasion and metastasis as well as poor prognosis in most common type of cancer [3]. However, there have been reports that decreased CD9 expression is associated with lymph node metastasis as well as distant metastasis in some cancers, which will be clarified in future studies [3].

Cancer morbidity and mortality are rapidly increasing around the world, and efforts have recently been made to find a biomarker that can provide reliable information for cancer patients [23, 24]. CD9, a tetraspanin protein, has various biological functions such as cellular adhesion, motility, cell growth, differentiation, signal transduction, and sperm-egg fusion [25]. CD9 plays an important role in many diseases, including viral and bacterial infections as well as cancer [25]. Previous researchers reported that CD9 is associated with cancer cell proliferation, survival, and metastasis, which is considered a potential biomarker for cancer prognosis [3, 23]. Nevertheless, studies have shown that CD9 expression is not consistent with the prognosis of cancer patients [25]. CD9 expression has been associated with favorable survival; CD9 expression has also been reported to suggest a poor prognosis in some cases. Researchers have reported that an increased CD9 expression is a favorable survival factor in patients with bladder [18], breast [8, 9], colorectal [10, 11], esophageal [12], head and neck [15, 16], gallbladder [13], lung [17], and pancreatic cancer [14], as well as malignant mesothelioma [19], acute myeloid leukemia [7], and follicular lymphoma [20]. On the contrary, other studies suggested that an increased CD9 expression was related to poor prognosis in patients with breast cancer [6, 21] and acute lymphoblastic leukemia [22]. Therefore, this meta-analysis was performed to better understand the association between CD9 expression and prognosis in cancer patients.

This meta-analysis was limited due to practical issues. First, the selection bias could not be completely ruled out, although studies were included in the analysis based on criteria. Second, standard criteria for CD9 expression were lacking among the included studies. This may have caused bias in our results. Finally, there was significant heterogeneity in this meta-analysis despite the multiple attempts to overcome heterogeneity including the random-effects model, subgroup analysis, and meta-regression. In particular, the CD9 detection method was presented as a cause of heterogeneity through meta-regression, which should be interpreted carefully because the experimental conditions of IHC, RT-PCR, and flow cytometry were not identified for each study included in the analysis. In future, more relevant studies will be required to evaluate the more important role of CD9 expression in human cancer.

## Conclusions

The current study is the first to systematically demonstrate the prognostic and clinicopathological significance of CD9 expression in cancer patients. In summary, an increased CD9 expression was associated with more favorable survival in cancer patients. This suggests that CD9 expression is a valuable survival factor in cancer patients. Additionally, our results revealed that increased CD9 expression was associated with increased OS in breast cancer and digestive system cancer and with increased DFS in head and neck cancer and leukemia/lymphoma. Moreover, CD9 expression was related to clinicopathological characteristics including overall stage.

## Supplementary Information

**Additional file 1. **Forest plot of the association between CD9 expression and clinicopathological characteristics in human cancers. **Figure S1.** age, **Figure S2**. patient’s sex, **Figure S3.** tumor size, **Figure S4**. tumor grade, **Figure S5**. tumor stage, **Figure S6**. lymph node metastasis, and **Figure S7.** overall stage.

## Data Availability

All the data used to support the findings of this study are included within the article.

## References

[1] Siegel R, Naishadham D, Jemal A (2012). Cancer statistics, 2012. CA Cancer J Clin.

[2] Fan H, Wang W, Yan J, Xiao L, Yang L (2018). Prognostic significance of CXCR7 in cancer patients: a meta-analysis. Cancer Cell Int.

[3] Murayama Y, Oritani K, Tsutsui S (2015). Novel CD9-targeted therapies in gastric cancer. World J Gastroenterol.

[4] Hori H, Yano S, Koufuji K, Takeda J, Shirouzu K (2004). CD9 expression in gastric cancer and its significance. J Surg Res.

[5] Houle CD, Ding X, Foley JF, Afshari CA, Barrett JC, Davis BJ (2002). Loss of expression and altered localization of KAI1 and CD9 protein are associated with epithelial ovarian cancer progression. Gynecol Oncol.

[6] Baek J, Jang N, Choi JE, Kim J, Bae YK (2019). CD9 expression in tumor cells is associated with poor prognosis in patients with invasive lobular carcinoma. J Breast Cancer.

[7] Touzet L, Dumezy F, Roumier C, Berthon C, Bories C, Quesnel B, Preudhomme C, Boyer T (2019). CD9 in acute myeloid leukemia: prognostic role and usefulness to target leukemic stem cells. Cancer Med.

[8] Miyake M, Nakano K, Itoi SI, Koh T, Taki T (1996). Motility-related protein-1 (MRP-1/CD9) reduction as a factor of poor prognosis in breast cancer. Cancer Res.

[9] Huang C, Kohno N, Ogawa E, Adachi M, Taki T, Miyake M (1998). Correlation of reduction in MRP-1/CD9 and KAI1/CD82 expression with recurrences in breast cancer patients. Am J Pathol.

[10] Kim KJ, Kwon HJ, Kim MC, Bae YK (2016). CD9 Expression in colorectal carcinomas and its prognostic significance. J Pathol Transl Med.

[11] Hashida H, Takabayashi A, Tokuhara T, Hattori N, Taki T, Hasegawa H, Satoh S, Kobayashi N, Yamaoka Y, Miyake M (2003). Clinical significance of transmembrane 4 superfamily in colon cancer. Br J Cancer.

[12] Shimada Y, Imamura M, Watanabe G, Uchida S, Harada H, Makino T, Kano M (1999). Prognostic factors of oesophageal squamous cell carcinoma from the perspective of molecular biology. Br J Cancer.

[13] Zou Q, Xiong L, Yang Z, Lv F, Yang L, Miao X (2012). Expression levels of HMGA2 and CD9 and its clinicopathological significances in the benign and malignant lesions of the gallbladder. World J Surg Oncol.

[14] Sho M, Adachi M, Taki T, Hashida H, Konishi T, Huang C, Ikeda N, Nakajima Y, Kanehiro H, Hisanaga M (1998). Transmembrane 4 superfamily as a prognostic factor in pancreatic cancer. Int J Cancer.

[15] Mhawech P, Dulguerov P, Tschanz E, Verdan C, Ares C, Allal AS (2004). Motility-related protein-1 (MRP-1/CD9) expression can predict disease-free survival in patients with squamous cell carcinoma of the head and neck. Br J Cancer.

[16] Erovic BM, Pammer J, Hollemann D, Woegerbauer M, Geleff S, Fischer MB, Burian M, Frommlet F, Neuchrist C (2003). Motility-related protein-1/CD9 expression in head and neck squamous cell carcinoma. Head Neck J Sci Special Head Neck.

[17] Higashiyama M, Taki T, Ieki Y, Adachi M, Huang CL, Koh T, Kodama K, Doi O, Miyake M (1995). Reduced motility related protein-1 (MRP-1/CD9) gene expression as a factor of poor prognosis in non-small cell lung cancer. Cancer Res.

[18] Mhawech P, Herrmann F, Coassin M, Guillou L, Iselin CE (2003). Motility-related protein 1 (MRP-1/CD9) expression in urothelial bladder carcinoma and its relation to tumor recurrence and progression. Cancer Interdiscipl Int J Am Cancer Soc.

[19] Amatya VJ, Takeshima Y, Aoe K, Fujimoto N, Okamoto T, Yamada T, Kishimoto T, Morimoto C, Inai K (2013). CD9 expression as a favorable prognostic marker for patients with malignant mesothelioma. Oncol Rep.

[20] Dong T, Liu Z, Zhao S, Hu C, Liu Y, Ma W, Zhang Q (2015). The expression of CD9 and PIK3CD is associated with prognosis of follicular lymphoma. J Cancer.

[21] Kwon HJ, Choi JE, Kang SH, Son Y, Bae YK (2017). Prognostic significance of CD9 expression differs between tumour cells and stromal immune cells, and depends on the molecular subtype of the invasive breast carcinoma. Histopathology.

[22] Liang P, Miao M, Liu Z, Wang H, Jiang W, Ma S, Li C, Hu R (2018). CD9 expression indicates a poor outcome in acute lymphoblastic leukemia. Cancer Biomark.

[23] Wang Y, Liu S, Tian Y, Wang Y, Zhang Q, Zhou X, Meng X, Song N (2018). Prognostic role of galectin-3 expression in patients with solid tumors: a meta-analysis of 36 eligible studies. Cancer Cell Int.

[24] Han L, Wang B, Wang R, Wang Z, Gong S, Chen G, Telemacque D, Feng Y, Xu W (2019). Prognostic and clinicopathological significance of long non-coding RNA PANDAR expression in cancer patients: a meta-analysis. Front Oncol.

[25] Brosseau C, Colas L, Magnan A, Brouard S (2018). CD9 tetraspanin: a new pathway for the regulation of inflammation?. Front Immunol.

