# Supplementary figures and images for: Increased CD9 expression predicts favorable prognosis in human cancers: a systematic review and meta-analysis
Source: Cancer Cell Int. 2021 Sep 7;21:472. doi: 10.1186/s12935-021-02152-y (PMC8422728; doi:10.1186/s12935-021-02152-y)

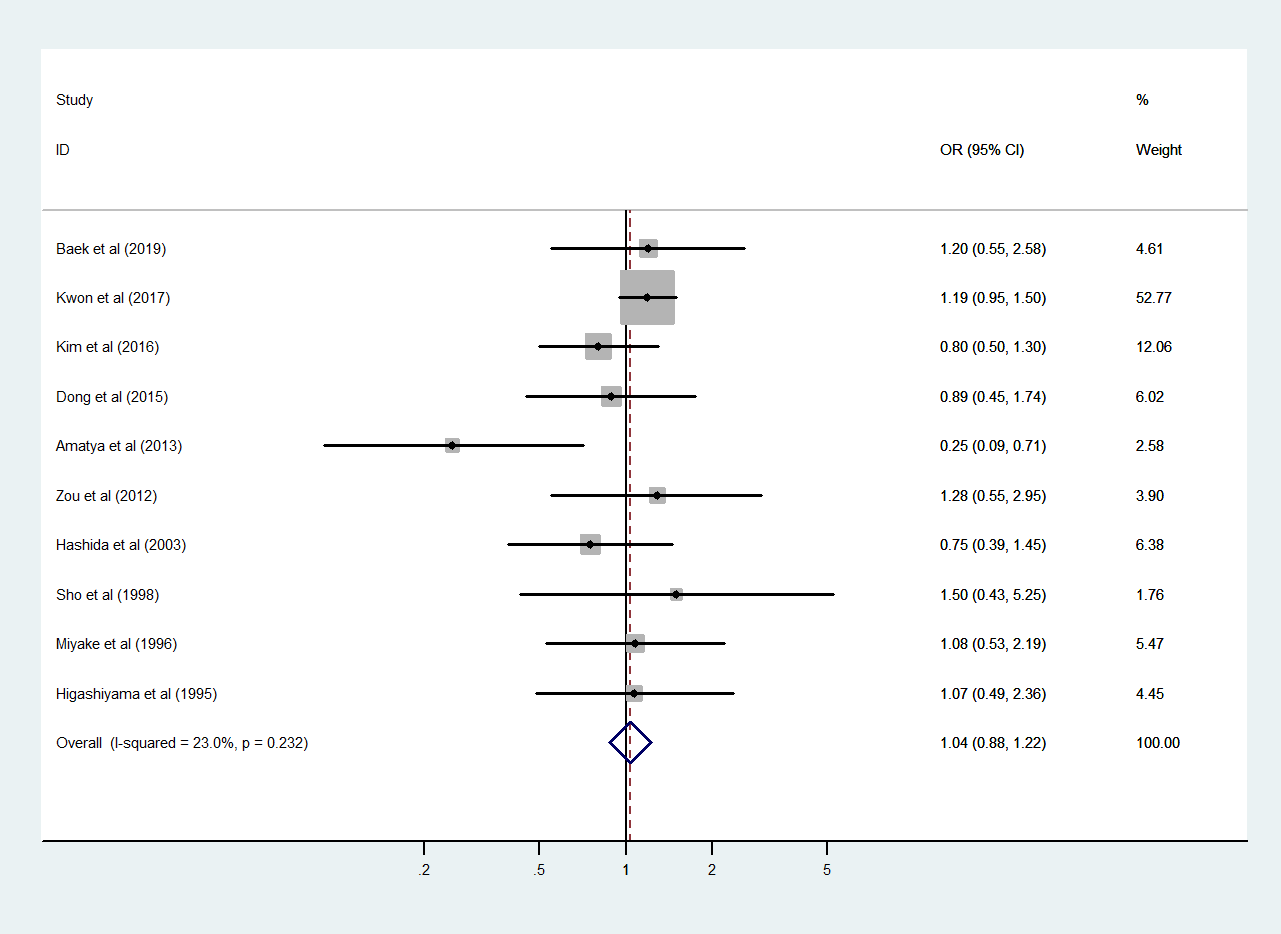

Supplement: Supplementary file 1 — Additional file 1. Forest plot of the association between CD9 expression and clinicopathological characteristics in human cancers. Figure S1. age, Figure S2. patient’s sex, Figure S3. tumor size, Figure S4. tumor grade, Figure S5. tumor stage, Figure S6. lymph node metastasis, and Figure S7. overall stage. [file 12935_2021_2152_MOESM1_ESM.zip › Figure S1.tif]

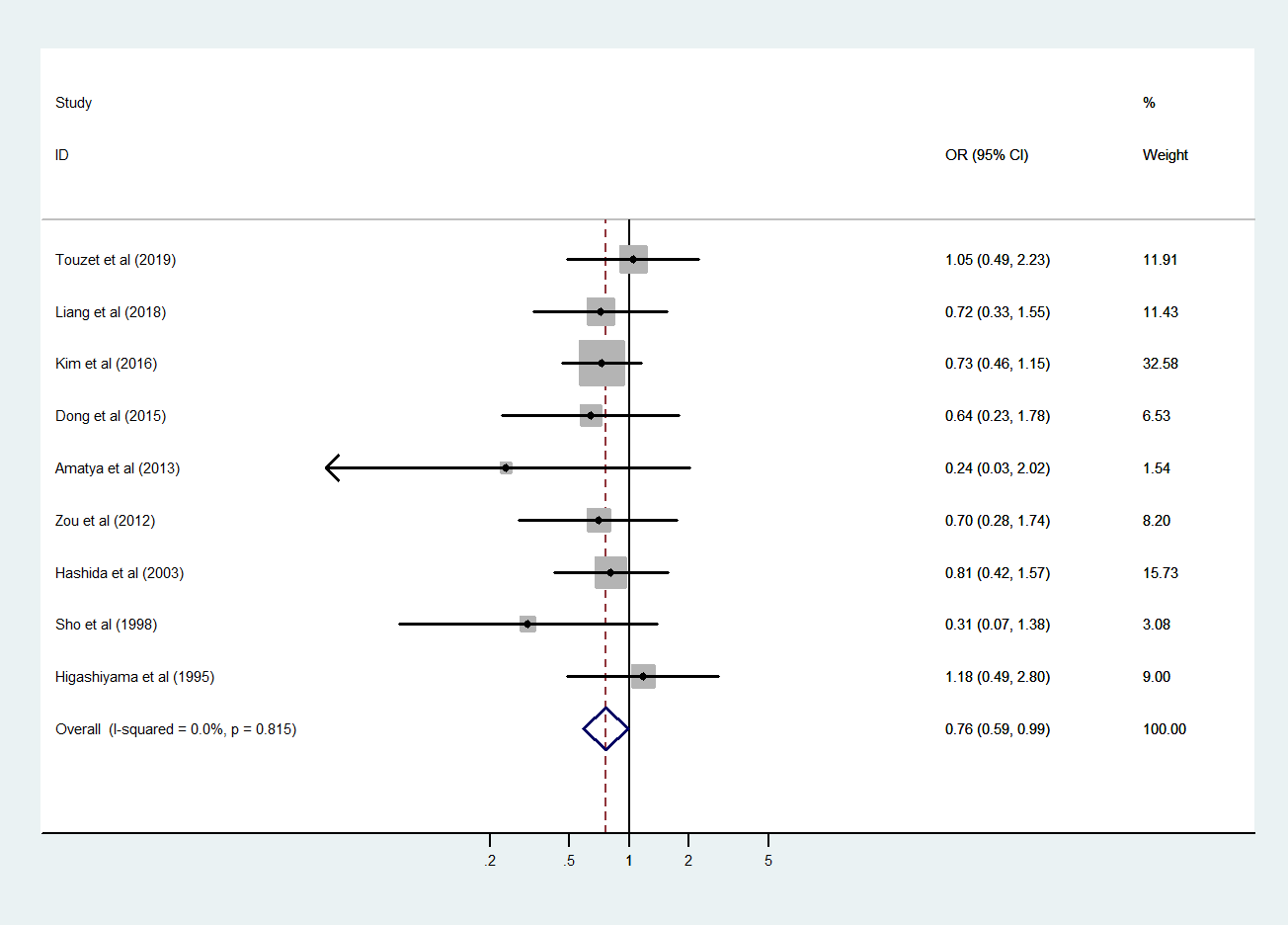

Supplement: Supplementary file 1 — Additional file 1. Forest plot of the association between CD9 expression and clinicopathological characteristics in human cancers. Figure S1. age, Figure S2. patient’s sex, Figure S3. tumor size, Figure S4. tumor grade, Figure S5. tumor stage, Figure S6. lymph node metastasis, and Figure S7. overall stage. [file 12935_2021_2152_MOESM1_ESM.zip › Figure S2.tif]

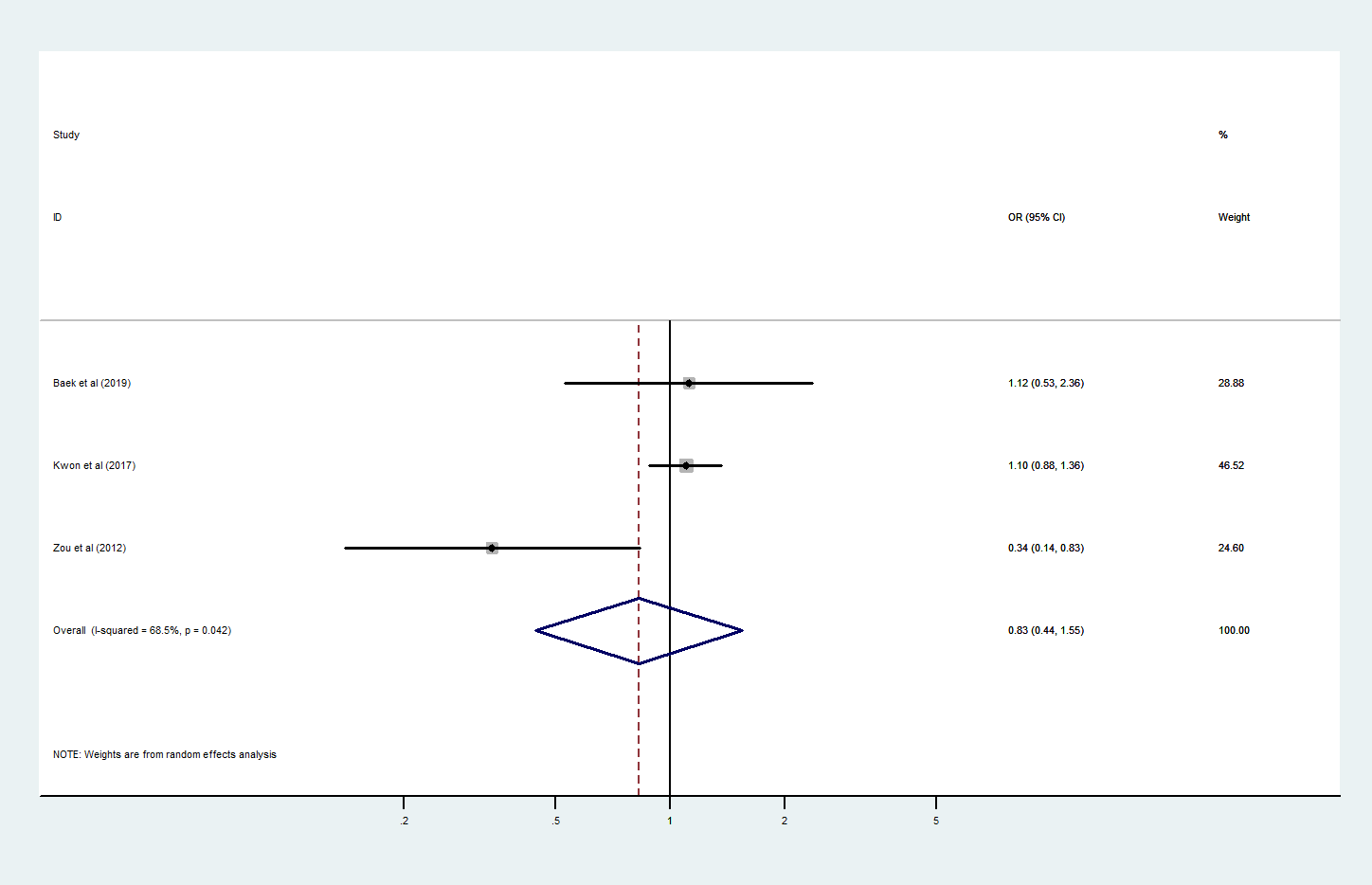

Supplement: Supplementary file 1 — Additional file 1. Forest plot of the association between CD9 expression and clinicopathological characteristics in human cancers. Figure S1. age, Figure S2. patient’s sex, Figure S3. tumor size, Figure S4. tumor grade, Figure S5. tumor stage, Figure S6. lymph node metastasis, and Figure S7. overall stage. [file 12935_2021_2152_MOESM1_ESM.zip › Figure S3.tif]

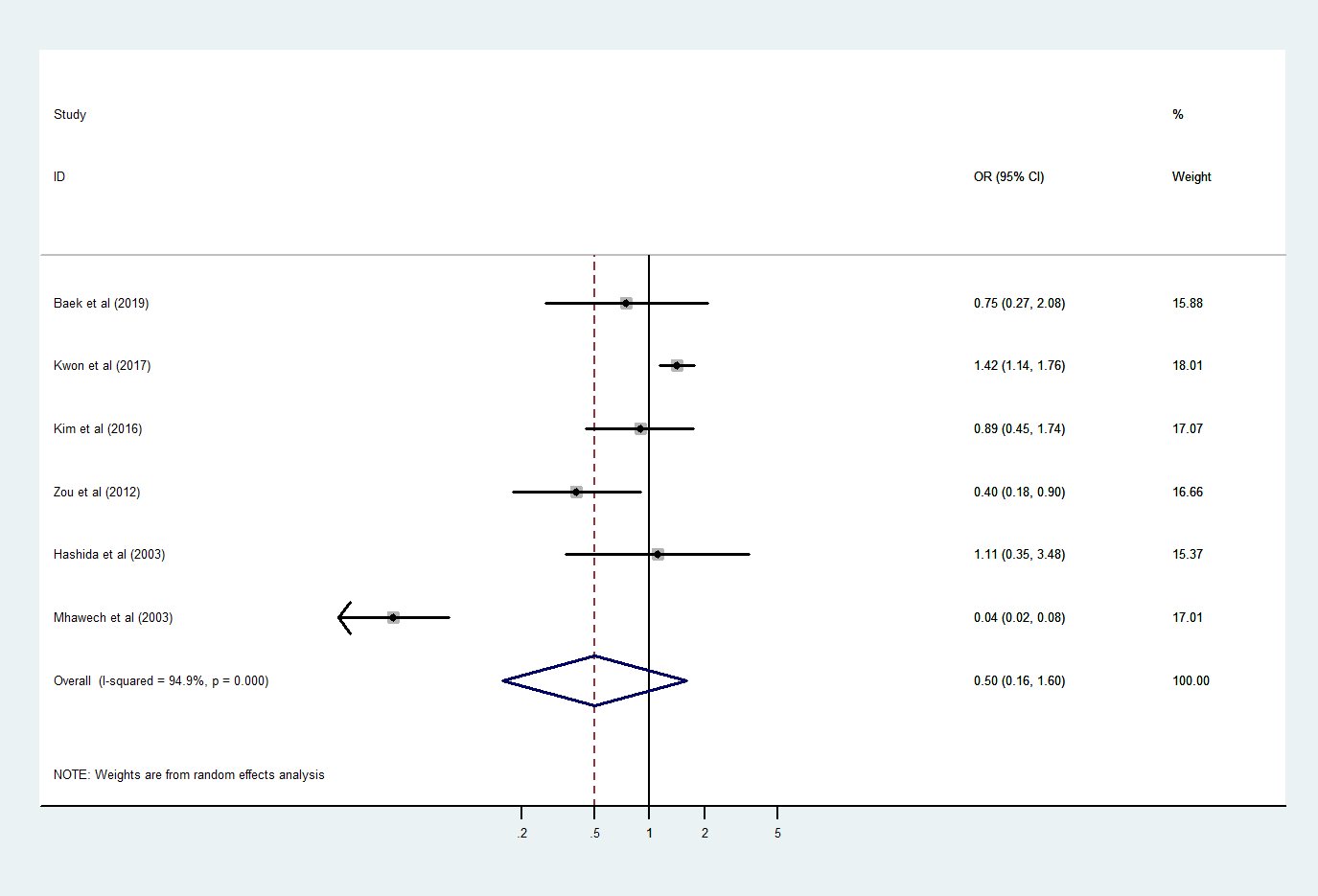

Supplement: Supplementary file 1 — Additional file 1. Forest plot of the association between CD9 expression and clinicopathological characteristics in human cancers. Figure S1. age, Figure S2. patient’s sex, Figure S3. tumor size, Figure S4. tumor grade, Figure S5. tumor stage, Figure S6. lymph node metastasis, and Figure S7. overall stage. [file 12935_2021_2152_MOESM1_ESM.zip › Figure S4.tif]

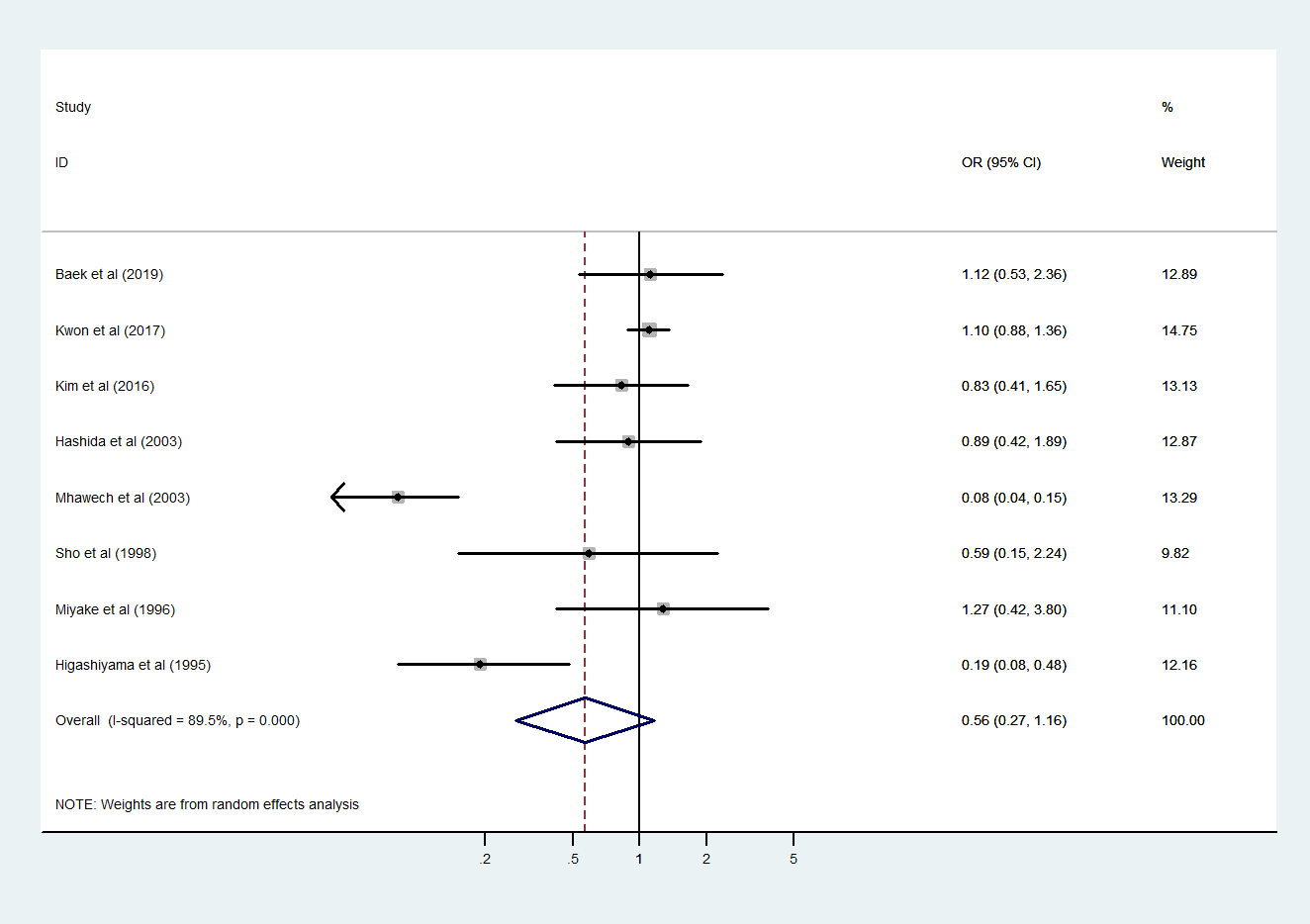

Supplement: Supplementary file 1 — Additional file 1. Forest plot of the association between CD9 expression and clinicopathological characteristics in human cancers. Figure S1. age, Figure S2. patient’s sex, Figure S3. tumor size, Figure S4. tumor grade, Figure S5. tumor stage, Figure S6. lymph node metastasis, and Figure S7. overall stage. [file 12935_2021_2152_MOESM1_ESM.zip › Figure S5.tif]

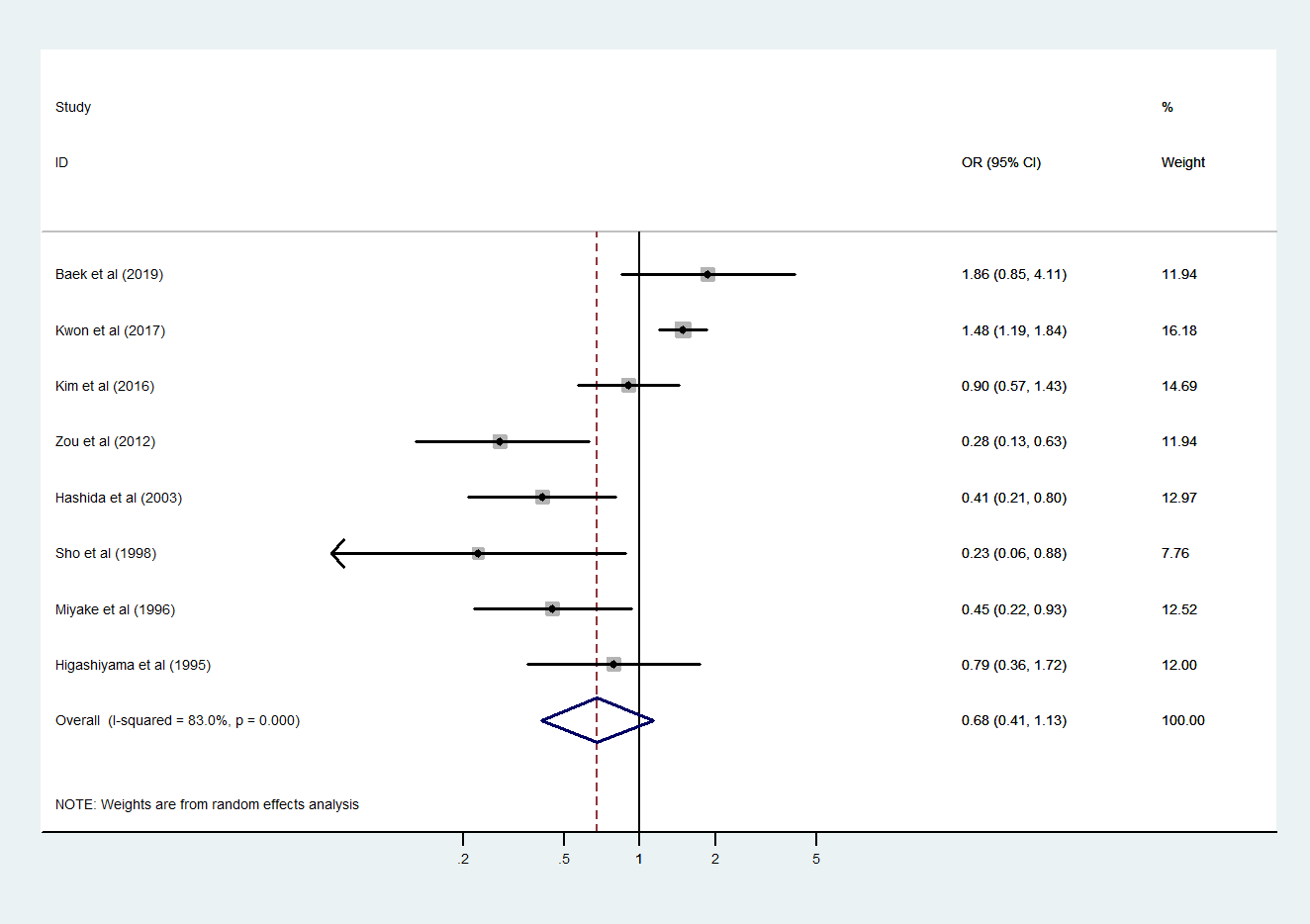

Supplement: Supplementary file 1 — Additional file 1. Forest plot of the association between CD9 expression and clinicopathological characteristics in human cancers. Figure S1. age, Figure S2. patient’s sex, Figure S3. tumor size, Figure S4. tumor grade, Figure S5. tumor stage, Figure S6. lymph node metastasis, and Figure S7. overall stage. [file 12935_2021_2152_MOESM1_ESM.zip › Figure S6.tif]

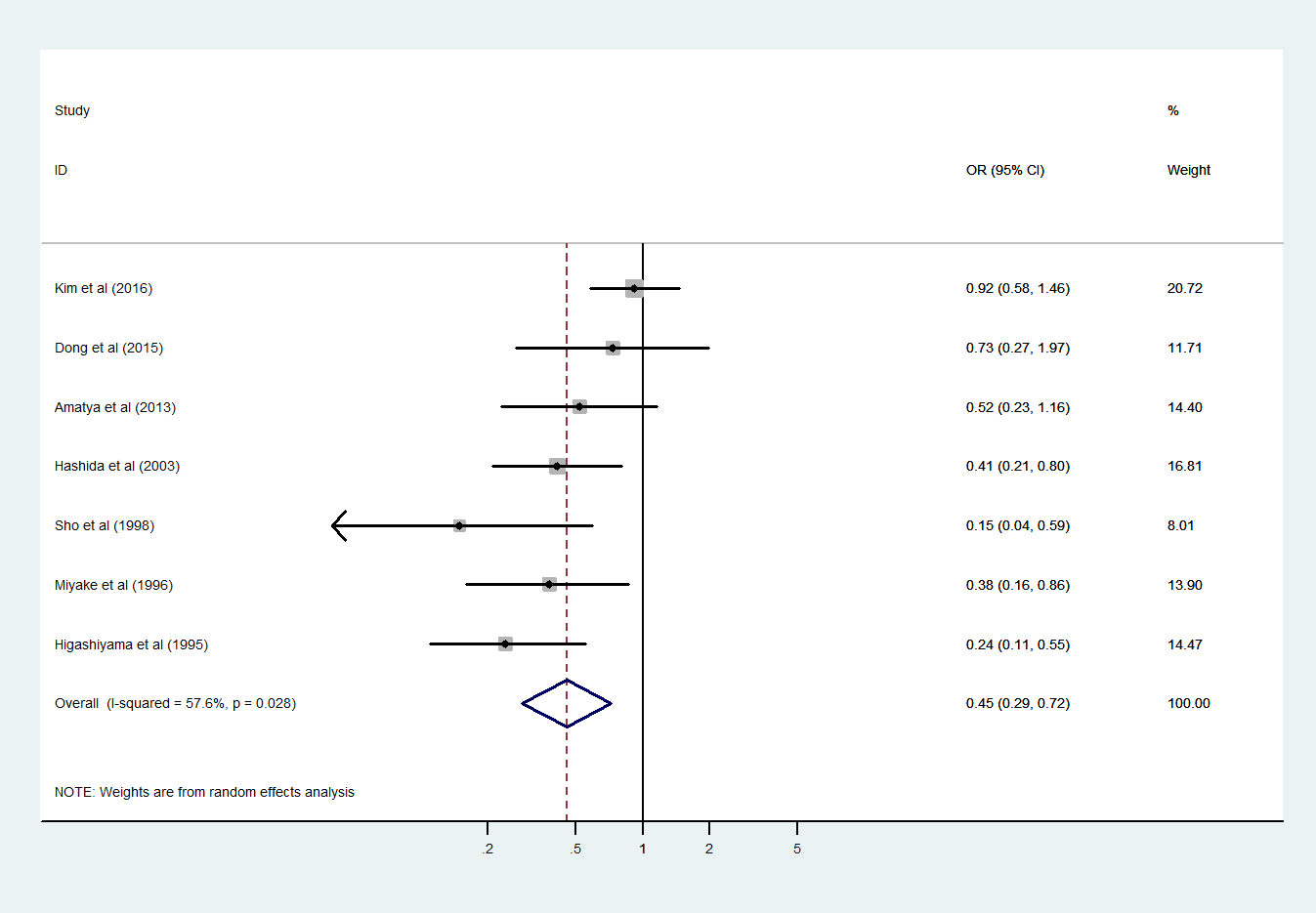

Supplement: Supplementary file 1 — Additional file 1. Forest plot of the association between CD9 expression and clinicopathological characteristics in human cancers. Figure S1. age, Figure S2. patient’s sex, Figure S3. tumor size, Figure S4. tumor grade, Figure S5. tumor stage, Figure S6. lymph node metastasis, and Figure S7. overall stage. [file 12935_2021_2152_MOESM1_ESM.zip › Figure S7.tif]
